# Hospitalized cardiovascular events in patients with diabetic macular edema

**DOI:** 10.1186/1471-2415-12-11

**Published:** 2012-07-12

**Authors:** Bao-Anh Nguyen-Khoa, Earl L Goehring, Winifred Werther, Anne E Fung, Diana V Do, Rajendra S Apte, Judith K Jones

**Affiliations:** 1 The Degge Group Ltd, 1616 North Fort Myer Drive, Suite 1430, Arlington, VA, 22209, USA; 2 Vertex Pharmaceuticals, Boston, MA, USA; 3 Pacific Eye Associates Ltd, San Francisco, CA, USA; 4 Wilmer Eye Institute, Johns Hopkins University School of Medicine, Baltimore, MD, USA; 5 Ophthalmology and Visual Sciences, Washington University, St Louis, MO, USA

## Abstract

**Background:**

Microvascular and macrovascular complications in diabetes stem from chronic hyperglycemia and are thought to have overlapping pathophysiology. The aim of this study was to investigate the incidence rate of hospitalized myocardial infarctions (MI) and cerebrovascular accidents (CVA) in patients with diabetic macular edema (DME) compared with diabetic patients without retinal diseases.

**Methods:**

This was a retrospective cohort study of a commercially insured population in an administrative claims database. DME subjects (n = 3519) and diabetes controls without retinal disease (n = 10557) were matched by age and gender. Healthcare claims were analyzed for the study period from 1 January 2002 to 31 December 2005. Incidence and adjusted rate ratios of hospitalized MI and CVA events were then calculated.

**Results:**

The adjusted rate ratio for MI was 2.50 (95% CI: 1.83-3.41, p < 0.001) for DME versus diabetes controls. Predictors of MI events were heart disease, history of acute MI, and prior use of antiplatelet or anticoagulant drugs. The adjusted rate ratio for CVA was 1.98 (95% CI: 1.39-2.83, p < 0.001) for DME versus diabetes controls. Predictors of CVA events were cardiac arrhythmia, Charlson comorbidity scores, history of CVA, hyperlipidemia, and other cerebrovascular diseases.

**Conclusion:**

Event rates of MI or CVA were higher in patients with DME than in diabetes controls. This study is one of few with sufficient sample size to accurately estimate the relationship between DME and cardiovascular outcomes.

## Background

Diabetic macular edema (DME) may occur at any stage of diabetic retinopathy (DR) and is the leading cause of moderate vision loss in adults of working age [1]. The prevalence of DME is about one-tenth that of background DR and one-third that of proliferative DR [2]. Microvascular complications, like DR and DME, are associated with progressive or uncontrolled diabetes. Cardiovascular events, such as myocardial infarctions (MI) or cerebrovascular accidents (CVA)/stroke, are known macrovascular complications of diabetes mellitus. Microvascular complications of diabetes are commonly diagnosed as eye diseases; however, pathologic changes to cardiac and cerebral circulation also occur [3].

The association between DR and cardiovascular outcomes has been extensively studied [4-8] and reviewed [9]. However, cardiovascular outcomes in DME patients have not been well examined; previously published studies with DME subjects did not have the power to characterize the relationship between DR and cardiovascular outcomes [6,10,11]. This report describes research in a large insured population with sufficient size to quantify and compare the incidence rates of MI or CVA in patients with DME against matched diabetes controls. The authors hypothesized that the presence of DME would be associated with higher rates of MI and CVA.

## Methods

### Database

The Ingenix LabRx Database™ (LabRx) contains administrative claims data for an employed, commercially insured population in the USA who are enrolled in 11 health insurance plans affiliated with the UnitedHealth Group. LabRx contains the covered healthcare experience of approximately 14 million patients annually. The health plans are geographically diverse, with the largest proportion of patients found in the Midwest of the USA. Complete pharmacy and medical claims data (including Medicare and Medicaid) were available for all the patients in this study.

The study involved the analysis of existing medical claims records that are recorded in such a manner that subjects cannot be identified directly or indirectly by the investigators. It met the exemption criteria for research activities not requiring institutional review under Title 45 Code of Federal Regulations Part 46.101 of the Basic HHS Policy for Protection of Human Research Subjects.

### Study design

A retrospective cohort study design was used to compare incidence rates of hospitalized MI and CVA in patients with DME and diabetic patients without retinal disease. Claims were analyzed for the 4-year period from 1 January 2002 to 31 December 2005.

### Study sample

#### DME patients

Patients were classified as having DME if they had medical claims for any combination of the following International Classification of Diseases, 9th Revision, Clinical Modification (ICD-9-CM) codes, in order of greater specificity:

1. 362.07 (DME) AND any code within the range of 362.01-362.06 (background DR). This 362.07 code combination was added for 2006 [12].

2. 362.83 (retinal edema) AND 250.5x (diabetes with ophthalmic manifestations) [2].

DME was classified using the ICD-9-CM code 362.01 from 2000 to 2005 [13]. During this period, this code was also used for DR. To test the ability of this code to accurately identify subjects with DME, three independent ophthalmologists were asked to evaluate 148 randomly selected administrative claims profiles of subjects identified with an ICD-9-CM code of 362.01 (background DR) and 250.5x (diabetes with ophthalmic manifestations) in the same eligibility period. An additional 44 profiles meeting definitions 1 and 2 were evaluated by each expert (five profiles with 362.07 and 39 profiles with 362.83). The percent agreement between all the reviewers was 2.7% for profiles with ICD-9-CM 362.01 for the definition of DME. Agreement for ICD-9-CM 362.07 and 362.83 was 100% and 90.7%, respectively. The ICD-9-CM code 362.01 in combination with 250.5x was judged to be non-specific for DME and therefore was excluded from the study.

A defined DME diagnosis had to appear at least once during a continuous eligibility period of > 183 days between 1 January 2002 and 31 December 2005. The index date was defined as the date of the first occurrence of ICD-9-CM codes 362.07 or 362.83 within the code combination sets above, following a minimum of at least 6 months (183 days) of baseline observation. Patients were included if they were at least 18 years old at the index date.

For an estimated sample of 3,000 subjects with DME, there was over 80% power to detect a hazard ratio of at least 1.25 with 95% confidence (i.e., 25% increased risk of a CV event) within either group.

#### Control patients

A set of control subjects with diabetes but without ophthalmic complications (ICD-9-CM 250.xx but excluding 250.5x), and without evidence of coding for DME during the study period were selected. Controls were excluded if coded for 362.x (other retinal disorders) or 379.23 (vitreous hemorrhage). Three controls were randomly selected without replacement for each identified DME patient. Patients were matched by gender and age using the following fixed ranges: 18–25, 26–30, 31–35, 36–40 years, and so on, through to 86+ years. Each control subject was assigned the same index date as the corresponding DME subject.

### Outcomes

Hospitalized MI and CVA events were identified using inpatient claims as defined by "Place of Service" field codes = 5 or 6 for "Inpatient hospital" and “Healthcare Cost Category” field code = 6. The Healthcare Cost Category was an additional claims field code used to ensure that outcomes were identified in an inpatient setting. The observation period for outcomes was from 1 July 2002 to 31 December 2005. Only the outcomes identified after a patient’s index date were counted as an event. Outcome events with admission and discharge dates that occurred on the same day were excluded because they are not reliable indicators of true events. Adjacent hospital claims with the same provider identification were counted as a single hospitalization if they were not separated by at least 1 day. Outcome events were identified in medical claims using the criteria of acute MI (ICD-9-CM 410.xx) or ischemic CVA (ICD-9-CM 433.x1, 434.x1, and 436.xx).

### Statistical analysis

For each patient, outcome events were recorded together with total observation time expressed to the nearest day. Patients were censored at the first occurrence of an event, end of eligibility, or end of study period. MI and CVA events were analyzed separately. All statistical calculations were made using SAS software version 9.1 (SAS Institute, Cary, NC, USA). Event rates and 95% confidence intervals (CI) were calculated for each outcome by age and gender as the number of events per 1000 person-years.

Cox regression was used to compare time to first-event rates between cohorts, with assumptions for proportional hazard satisfied. Covariates were added to the model to adjust for the presence of known risk factors for MI and CVA during the 183 days of continuous eligibility preceding the index date. Covariates included history of MI or CVA prior to index, congestive heart failure (inpatient only), cardiac arrhythmia (inpatient only), other cerebrovascular diseases, hypertension, hyperlipidemia, heart disease (coronary artery disease), angina, and the prior use of prescription anticoagulant or antiplatelet drugs. In addition, a modified Charlson Comorbidity Index (CCI) score was used as a covariate for the burden of comorbidities between the cohorts. And diseases comprising the CCI were mapped to ICD-9-CM codes [14-16]. In this study, codes for the outcomes of MI or CVA were excluded from the index.

## Results

There were 3519 patients identified with DME and 10557 matched diabetic controls with no claims for retinal disorders. Enrollment duration and follow-up time were similar between the groups (Table 1). The mean age for DME patients was 58 years with slightly more men than women. Modified CCI scores were generally higher in the DME group where more subjects had scores of ≥ 2 (56.6% vs. 23.0%), thus indicating a greater disease burden (Table 1).

**Table 1:** Demographic of subjects with DME and age/gender matched diabetes controls, 1 July 2002 to 31 December 2005

	**DME subjects**		**Diabetes controls**	
	**n**	**%**	**n**	**%**
**All subjects**	**3519**	**100**	**10557**	**100**
Female	1645	46.7	4935	46.7
Male	1874	53.3	5622	53.3
**Age groups**				
18-29	49	1.4	157	1.5
30-39	217	6.2	591	5.6
40-49	517	14.7	1522	14.4
50-59	1144	32.5	3271	31.0
60-69	937	26.6	2971	28.1
70-79	516	14.7	1521	14.4
80-89	139	3.9	524	5.0
Mean	58	-	59	-
Median	58	-	59	-
SD	12	-	12	-
**Region**				
Midwest	1449	41.2	3485	33.0
Northeast	353	10.0	1184	11.2
South	1430	40.6	4803	45.5
West	287	8.2	1085	10.3
Charlson comorbidity index*				
0	294	8.4	3355	31.8
1	1235	35.1	4772	45.2
2+	1990	56.6	2430	23.0
**Eligibility duration**	**Days**	**Years**	**Days**	**Years**
Mean	1116	3.1	1085	3.0
Median	1218	3.3	1186	3.2
SD	371	1.0	398	1.1
**Follow-up time**				
Mean	504	1.4	487	1.3
Median	438	1.2	417	1.1
SD	356	1.0	362	1.0

*Chi-square, p < 0.0001.

DME = diabetic macular edema; SD = standard deviation.

### Incidence of MI

The rate of MI events was higher in the DME cohort than in controls (Table 2). There were 94 subjects (2.7%) that developed a MI in the DME group for a rate of 19.7 MI events/1000 person-years (95% CI: 15.7-23.6). In the control group, there were 96 MIs (0.9%), for a rate of 6.9 MI events/1000 person-years (95% CI: 5.5-8.2). The rates of MI were higher in DME patients for both genders and, within both groups, rates were slightly higher in men than in women. There was an age-related increase in event rates in both cohorts, with the DME cohort higher in each age bracket (Table 2).

**Table 2:** Incidence of AMI in subjects with DME and age/gender matched diabetes controls*, 1 July 2002 to 31 December 2005

			**DME Subjects**					**Diabetes Controls**		
**Overall**	**n**	**Person-years**	**AMI events**	**rate/1000 PY**	**95% CI**	**n**	**Person-years**	**AMI events**	**rate/1000 PY**	**95% CI**
**All subjects**	**3519**	4778.5	**94**	**19.7**	**15.7 - 23.6**	**10557**	13981.5	**96**	**6.9**	**5.5 - 8.2**
Females	1645	2220.9	38	17.1	11.7 - 22.5	4935	6502.4	40	6.2	4.3 - 8.1
Males	1874	2557.6	56	21.9	16.2 - 27.5	5622	7479.1	56	7.5	5.5 - 9.4
			**DME Subjects**					**Diabetes Controls**		
**Age (years)**	**n**	**Person-years**	**AMI events**	**rate/1000 PY**	**95% CI**	**n**	**Person-years**	**AMI events**	**rate/1000 PY**	**95% CI**
18-29	49	58.5	0	0.0	0.0 - 0.0	157	178.5	0	0.0	0.0 - 0.0
30-39	217	280.9	2	7.1	0.0 - 16.9	591	772.9	3	3.9	0.0 - 8.3
40-49	517	717.6	9	12.5	4.4 - 20.7	1522	1969.7	4	2.0	0.0 - 4.0
50-59	1144	1531.4	28	18.3	11.6 - 25.0	3271	4293.2	17	4.0	2.1 - 5.8
60-69	937	1245.5	28	22.5	14.3 - 30.7	2971	3777.9	31	8.2	5.3 - 11.1
70-79	516	745.0	19	25.5	14.2 - 36.8	1521	2229.4	28	12.6	8.0 - 17.2
80-89	139	199.6	8	40.1	13.1 - 67.0	524	759.9	13	17.1	7.9 - 26.3

*Diabetes controls - diabetes subjects without ophthalmic manifestations, retinal disorders, or vitreous hemorrhage.

DME = diabetic macular edema; AMI = acute myocardial infarction; CVA = cerebrovascular accident; PY = person-years;

CI = confidence interval.

After adjustment for risk factors and CCI score, the rate of MI events was significantly higher in the DME group than in controls (adjusted HR 2.50, 95% CI: 1.83-3.41, p < 0.001). The presence of heart disease, history of MI, and prior use of antiplatelet or anticoagulant drugs were significant positive predictors of MI in the regression model (Table 3).

**Table 3:** Adjusted HRs for development of AMI in subjects with DME compared with age/gender matched diabetes controls*, 1 July 2002 to 31 December 2005

**Reference exposure**	**HR**	**95% CI**	**p-value**
**DME versus diabetes controls**	**2.50**	**1.83 – 3.41**	**< 0.001**
**Covariates†**			
Angina	1.00	0.59 - 1.71	1.00
Cardiac arrhythmia	1.19	0.75 - 1.87	0.47
Charlson comorbidity index	0.98	0.88 - 1.08	0.63
Congestive heart failure	1.57	0.98 - 2.52	0.06
**Heart disease**	**2.45**	**1.72 - 3.48**	**< 0.001**
**History of AMI**	**2.62**	**1.35 - 5.07**	**0.004**
History of CVA	1.37	0.71 - 2.66	0.35
Hyperlipidemia	0.75	0.55 - 1.01	0.06
Hypertension	0.92	0.67 - 1.27	0.62
Other cerebrovascular disease	1.47	0.68 - 3.19	0.33
**Prior anti-platelet or anticoagulant**	**1.93**	**1.28 - 2.89**	**0.002**

*Diabetes controls - diabetes subjects without ophthalmic manifestations, retinal disorders, or vitreous hemorrhage.

† All covariates were included in the regression model; bolded text indicates statistical signficance.

DME = diabetic macular edema; AMI = acute myocardial infarction; CVA = cerebrovascular accident; CI = confidence interval; HR = hazard ratios.

### Incidence of CVA

The rate of CVA events was also higher in the DME group than in controls (Table 4). There were 66 subjects (1.9%) that developed a CVA in the DME group, for a rate of 13.8 CVA events/1000 person-years (95% CI: 10.5-17.0). In the control group, there were 75 CVAs (0.7%), for a rate of 5.4 events/1000 person-years (95% CI: 4.1-6.6).

**Table 4:** Incidence of CVAs in subjects with DME and age/gender matched controls*, 1 July 2002 to 31 December 2005

			**DME Subjects**					**Diabetes Controls**		
**Overall**	**n**	**Person-years**	**AMI events**	**rate/1000 PY**	**95% CI**	**n**	**Person-years**	**AMI events**	**rate/1000 PY**	**95% CI**
**All subjects**	**3519**	4778.5	**66**	**13.8**	**10.5 - 17.0**	**10557**	13981.5	**75**	**5.4**	**4.1 - 6.6**
Females	1645	2220.9	38	17.1	11.7 - 22.5	4935	6502.4	38	5.8	4.0 - 7.7
Males	1874	2557.6	28	10.9	6.9 - 14.9	5622	7479.1	37	4.9	3.3 - 6.5
			**DME Subjects**					**Diabetes Controls**		
**Age (years)**	**n**	**Person-years**	**AMI events**	**rate/1000 PY**	**95% CI**	**n**	**Person-years**	**AMI events**	**rate/1000 PY**	**95% CI**
18-29	49	58.5	0	0.0	0.0 - 0.0	157	178.5	0	0.0	0.0 - 0.0
30-39	217	280.9	1	3.6	0.0 - 10.5	591	772.9	0	0.0	0.0 - 0.0
40-49	517	717.6	8	11.2	3.5 - 18.8	1522	1969.7	1	0.5	0.0 - 1.5
50-59	1144	1531.4	18	11.7	6.3 - 17.1	3271	4293.2	17	4.0	2.1 - 5.8
60-69	937	1245.5	21	16.7	9.6 - 23.8	2971	3777.9	18	4.7	2.6 - 6.9
70-79	516	745.0	14	18.8	9.1 - 28.5	1521	2229.4	20	9.0	5.1 - 12.9
80-89	139	199.6	4	19.6	0.7 - 38.6	524	759.9	19	25.1	14.0 - 36.3

*Diabetes controls - diabetes subjects without ophthalmic manifestations, retinal disorders, or vitreous hemorrhage.

DME = diabetic macular edema; CVA = cerebrovascular accident; PY = person-years; CI = confidence interval.

The rates of CVA were also higher in the DME group for both genders. In particular, the crude risk of stroke in men with DME was 2-times higher than in the control group, and nearly 3-times higher in women with DME. Again, there was an age-related increase in rates of CVA in both groups, with DME higher in each age bracket except for the 80–89 years group (Table 4).

After adjustment for risk factors, the rate of CVA events after the index date was significantly higher in the DME group than in the control group (adjusted HR 1.98, 95% CI: 1.39-2.83, p < 0.001). Positive predictors in the regression model included cardiac arrhythmia, CCI score, history of CVA, hyperlipidemia, and other cerebrovascular diseases. The presence of hyperlipidemia was inversely associated with the development of CVA (Table 5).

**Table 5:** Adjusted HRs for development of CVAs in subjects with DME compared with age/gender matched diabetes controls*, 1 July 2002 to 31 December 2005

**Reference exposure**	**HR**	**95%** **CI**	**p-value**
**DME versus diabetes controls**	**1.98**	**1.39 – 2.83**	**< 0.001**
**Covariates†**			
Angina	0.71	0.32 - 1.58	0.40
**Cardiac arrhythmia**	**1.88**	**1.12 - 3.16**	**0.02**
**Charlson comorbidity index**	**1.13**	**1.02 - 1.24**	**0.02**
Congestive heart failure	0.94	0.51 - 1.72	0.84
Heart disease	1.52	0.99 - 2.32	0.06
History of AMI	0.91	0.31 - 2.69	0.87
**History of CVA**	**2.40**	**1.26 - 4.59**	**0.01**
**Hyperlipidemia**	**0.63**	**0.44 - 0.91**	**0.01**
Hypertension	1.18	0.81 - 1.70	0.39
**Other cerebrovascular disease**	**2.20**	**1.03 - 4.71**	**0.04**
Prior anti-platelet or anticoagulant	1.07	0.62 - 1.83	0.81

*Diabetes controls - diabetes subjects without ophthalmic manifestations, retinal disorders, or vitreous hemorrhage.

† All covariates were included in the regression model; bolded text indicates statistical signficance.

DME = diabetic macular edema; AMI = acute myocardial infarction; CVA = cerebrovascular accident; CI = confidence interval; HR = hazard ratios.

## Discussion

In this analysis of a large insured population of patients with diabetes, the presence of DME is associated with a higher incidence of MI and CVA. Adjusted estimates show a 2.5-fold higher risk of MI and a 2-fold higher risk of CVA in patients with DME compared with diabetic patients without retinal disorders. Incident stroke appeared to be higher in women than men in either group, which is supported by data from other studies [17].

Earlier studies have shown that the presence of diabetic retinopathy is a predictor of cardiovascular morbidity and mortality [4-8,10]. The Diabetes Control and Complications Trial reported that tight control of diabetes with insulin reduced the 36-month risk of progression to retinopathy by 76% (95% CI: 62-85%) [18]. In the case of type 2 diabetes, the UK Prospective Diabetes Study showed that for every 1% decrease in hemoglobin A1c (HbA1c), there was a 35% reduction in the risk of microvascular complications [19].

Other studies have reported cardiovascular outcomes in DME that appear to support the findings of this study. The Wisconsin Epidemiologic Study of Diabetic Retinopathy (WESDR) examined the relationship between several diabetic eye diseases and mortality over a 16-year period [10]. Patient cohorts with type I or II diabetes with disease onset before and after the age of 30 years were separately examined. In "younger-onset" diabetes subjects with DME (n = 92), there was a non-significant decrease in likelihood of death from ischemic heart disease compared with similar subjects without DME (adjusted HR 0.84, 95% CI: 0.43-1.66). In "older-onset" DME subjects (n = 136), there was a non-significant increase in the likelihood of death from either heart disease or stroke (adjusted HR 1.10, 95% CI: 0.76-1.58 and 1.17, 95% CI: 0.65-2.10, respectively) [10]. The design and outcome measures of the WESDR study make its results difficult to directly compare with the results of the current study.

Cheung and colleagues examined the relationship between DR and cardiovascular events in two studies using data from The Atherosclerosis Risk in Communities study [6,11]. In the first, investigators performed a prospective cohort study on 1617 patients with diabetes to quantify the relationship between DR and ischemic stroke. In subgroup analyses, there was a non-significant increase in the risk of stroke in subjects with DME (n = 23, adjusted HR 1.40, 95% CI: 0.54-3.65) [6]. Separately, Cheung et al. also found that the presence of DR was associated with 2-times the risk of incident coronary heart disease events (adjusted HR 2.07, 95% CI: 1.38-3.11) [11]. However, in subgroup analyses, there was no change in the risk of coronary events in subjects with DME (n = 81, adjusted HR 1.00, 95% CI: 0.56-1.79). With regards to DR, the authors concluded that their findings supported the role of microvascular disease in the pathogenesis of heart disease in diabetes. Unfortunately, neither study was sufficiently powered with DME patients to detect a significant difference.

Several limitations related to all administrative claims studies are worth mentioning. Previous studies have shown that diabetes duration, elevated HbA1c levels and uncontrolled hypertension are common risk factors for DR [20] and cardiovascular events [21]. As with most claims studies, the onset date of a chronic disease is difficult to determine. The incidence of DME increases with longer duration of type I diabetes [20]. Diabetes duration is a predictor for the development of retinopathy and DME [22,23] and is not measurable in this study. However, the cohorts in this study were age-matched, which should minimize any differences between the age of diabetes onset and therefore differences in the duration of diabetes. This does not fully account for residual confounding influenced by duration. In this study, HbA1c results were available for 25% of DME patients and 19% of controls with mean values of 7.3% and 7.0%, respectively (a sample of patients without diabetes had a mean value of 5.0%). The American Diabetes Association’s standards of care establish a HbA1c goal of 7%, so the subset of patients in this study appears to be within reasonable glycemic control. If this is the case, the difference in the available data for this study does not appear to be clinically significant, but glycemic control was not measured in all patients and therefore was not adjusted in the regression model. Blood pressure data were not available to evaluate the effect of uncontrolled hypertension, but overall hypertension diagnoses were adjusted in the analysis. In a *post-hoc* analysis of untreated hypertension within each cohort, fewer DME patients had hypertension without antihypertensive therapy than did controls (9.3% vs. 12.7%). Thus, uncontrolled hypertension alone is unlikely to account for the difference in cardiovascular events.

Recently, the code combination 362.53 (cystoid macular edema) plus 250.xx (diabetes mellitus) was found to have high sensitivity and specificity for DME in a regional study of 22 ophthalmology practices [24]. The current study did not use cystoid macular edema as a definition of DME in the inclusion criteria; therefore, additional subjects with DME may have been excluded. It is not known how the inclusion of this additional code would have affected the results. Lastly, administrative claims cannot differentiate between DME and clinically significant DME; any divergence in the rates of cardiovascular events in these clinical subgroups is not known.

One implication of quantifying baseline rates of cardiovascular events in DME is the advent of agents that locally suppress vascular endothelial growth factors (VEGF) in the retina but that may also have systemic effects. Studies have shown that VEGF inhibitors may be effective in treating DME [25]. However, if VEGF inhibition reaches the systemic circulation, it may play a role in potentiating cardiovascular complications in diabetes [26]. As they are adopted into clinical practice, additional population research will be needed to evaluate the long-term safety of advanced drugs that target the microvascular complications of diabetes.

## Conclusions

To our knowledge, previous studies have lacked sufficient power to estimate the risk of hospitalized MI or CVA in patients with DME. In this report, both MI and CVA events were significantly higher in patients with DME than in diabetic patients without retinal disease. Microvascular and macrovascular complications of diabetes are thought to share pathogenic mechanisms. This presents an opportunity for risk communication between ophthalmologists and their diabetic patients, particularly if a diagnosis of DME is made. Informed by this analysis, future studies should be designed that address unmeasured confounders to better characterize the risk of cardiovascular events in the DME population.

## Misc

This submission has not been previously published elsewhere and is not concurrently under consideration by any other publication.

## Competing interests

Genentech Inc. provided financial support to The Degge Group, Ltd. for this study. BN, ELG, and JKJ are employees of The Degge Group, Ltd. No additional funding was received. Neither Genentech nor any representative of the sponsor took part in the collection, management, or analysis of the data. At the time of research, Dr. Werther was an employee of Genentech Inc., her role is described in the authors' contribution section. The decision to publish was made jointly by Genentech and Degge prior to the conduct of this research. The preparation of this manuscript was included in the funding prior to the conduct of research. At the time of research, Dr. Winifred Werther was a senior epidemiologist at the study sponsor, Genentech. Dr Rajendra Apte and Dr Anne Fung have served as consultants and speakers for Genentech. Dr Diana Do and Dr Anne Fung have received clinical research funding from Genentech. Dr. Rajendra Apte has also consulted for Eyetech and Allergan, and has equity ownership in Opthotech. Dr Judith Jones is President of The Degge Group, and Dr Bao-Anh Nguyen-Khoa and Mr Earl Goehring are employees of The Degge Group. The Degge Group had full access to all of the data in the study and takes responsibility for the integrity of the data and the accuracy of the data analyses.

## Authors’ contribution

BN conceived the study and study design, was involved in the data collection and analysis, and drafted/revised the manuscript. ELG was involved in the study design, data procurement, data collection, and provided assistance in drafting the manuscript, as well as review and critical revision of the subsequent drafts. WW was involved in provision of the study funding, conception of the study, the study design, and provided review and interpretation in the during manuscript development. WW was not involved in the collection, management, or analysis of the data. AF was involved in the study design and statistical analysis, provided interpretation and critical review of intellectual content for the draft manuscript. DD was involved in the study design, provided critical review and interpretation of intellectual content for the draft manuscript. RA was involved in the study design, and provided interpretation and critical review of intellectual content for the draft manuscript. JKJ was involved in obtaining the study funding, conception and study design, and provided interpretation and critical review of intellectual content for the draft manuscript. All authors read and approved the final manuscript.

## Pre-publication history

The pre-publication history for this paper can be accessed here:

http://www.biomedcentral.com/1471-2415/12/11/prepub
